# Activation of the EGF Receptor by Ligand Binding and Oncogenic Mutations: The “Rotation Model”

**DOI:** 10.3390/cells6020013

**Published:** 2017-06-02

**Authors:** Endang R. Purba, Ei-ichiro Saita, Ichiro N. Maruyama

**Affiliations:** Information Processing Biology Unit, Okinawa Institute of Science and Technology Graduate University, Okinawa 904-0495, Japan; endang.purba@oist.jp (E.R.P.); eiichiro.saita@oist.jp (E.-i.S.)

**Keywords:** cancer, cell-surface receptor, EGFR, molecular mechanism, phosphorylation, receptor tyrosine kinase, transmembrane signal transduction

## Abstract

The epidermal growth factor receptor (EGFR) plays vital roles in cellular processes including cell proliferation, survival, motility, and differentiation. The dysregulated activation of the receptor is often implicated in human cancers. EGFR is synthesized as a single-pass transmembrane protein, which consists of an extracellular ligand-binding domain and an intracellular kinase domain separated by a single transmembrane domain. The receptor is activated by a variety of polypeptide ligands such as epidermal growth factor and transforming growth factor α. It has long been thought that EGFR is activated by ligand-induced dimerization of the receptor monomer, which brings intracellular kinase domains into close proximity for *trans*-autophosphorylation. An increasing number of diverse studies, however, demonstrate that EGFR is present as a pre-formed, yet inactive, dimer prior to ligand binding. Furthermore, recent progress in structural studies has provided insight into conformational changes during the activation of a pre-formed EGFR dimer. Upon ligand binding to the extracellular domain of EGFR, its transmembrane domains rotate or twist parallel to the plane of the cell membrane, resulting in the reorientation of the intracellular kinase domain dimer from a symmetric inactive configuration to an asymmetric active form (the “rotation model”). This model is also able to explain how oncogenic mutations activate the receptor in the absence of the ligand, without assuming that the mutations induce receptor dimerization. In this review, we discuss the mechanisms underlying the ligand-induced activation of the preformed EGFR dimer, as well as how oncogenic mutations constitutively activate the receptor dimer, based on the rotation model.

## 1. Introduction

**Figure 1:**
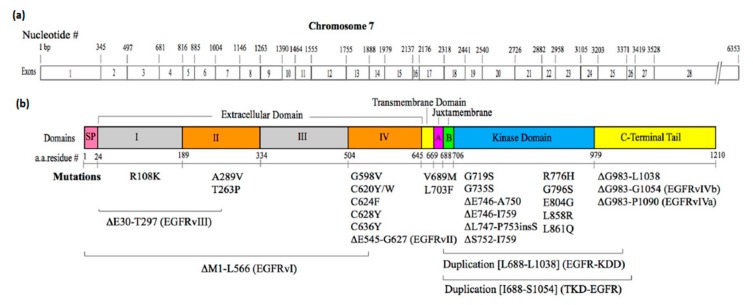
Schematic diagrams of the domains of EGFR and oncogenic mutation sites. (**a**) Exons encoding the human EGFR protein (EMBL/GenBank Accession No. AF288738; NCBI Accession No. NM_005228.4). Nucleotide sequence numbers of exon boundaries are shown above the exon diagram; (**b**) Domain structure of EGFR. Oncogenic mutation sites are also shown below the structure. Amino acid residue numbers (a.a. residue #), including the signal peptide sequences, are also shown below the domain diagram. Mutation sites are shown using a.a. residue numbers, including the signal peptide sequences.

The epidermal growth factor receptor (EGFR) is a member of the ErbB receptor family, which is a member of the receptor tyrosine kinase superfamily. The ErbB receptor family consists of EGFR (also known as ErbB1 or HER1), ErbB2 (Neu or HER2), ErbB3 (HER3), and ErbB4 (HER4). EGFR is involved in a variety of cellular processes including cell proliferation, motility, survival, and differentiation, and is essential for normal animal development [1,2,3,4]. The aberrant activation of EGFR is implicated in a variety of human cancers [5]. The receptor is activated by the binding of various ligands including epidermal growth factor (EGF), transforming growth factor α (TGFα), amphiregulin (AREG), epigen, β-cellulin, heparin-binding EGF (HB-EGF), and epiregulin [6,7]. EGFR is a single-pass transmembrane protein, consisting of an extracellular domain, a transmembrane domain, a juxtamembrane (JM) segment, a kinase domain, and a C-terminal regulatory tail (Figure 1) [8,9]. Upon ligand binding, the C-terminal tail becomes tyrosine-phosphorylated, and mediates interactions between the receptor and downstream effectors such as Shc1 and Grb2 [10]. The extracellular ligand-binding domain of EGFR contains four distinct Subdomains I–IV [11,12,13,14,15]. Both Subdomains I (also known as L1) and III (or L2) have a β-helix solenoid structure, which is related to the leucine-rich repeat superfamily [16], and are responsible for ligand binding through simultaneous contact to the same bound ligand. Subdomains II (or CR1) and IV (or CR2) are both cysteine-rich regions with disulfide bonds similar to those seen in laminin and the tumor necrosis factor receptor [17]. The intracellular domain has the tyrosine kinase domain flanked by the intracellular JM segment and the C-terminal tail. The intracellular domain of EGFR has 20 tyrosine residues, 12 of which are known to be phosphorylated [18,19,20]. These phosphotyrosine residues bind soluble or membrane-anchored effector proteins that are recruited upon receptor activation [21,22,23]. EGFR activates several downstream signaling cascades, which include pathways mediated by Ras/Raf/MAP kinase, phosphoinositide-3-kinase (PI3K)/Akt, and phospholipase Cγ [10,24].

Based on unliganded monomeric and dimeric structures, two mutually exclusive models, the “ligand-induced dimerization model” and the “rotation model”, have been proposed for the activation of EGFR by ligand binding. According to the “ligand-induced dimerization model”, EGFR is activated by the ligand-induced dimerization of the receptor monomer, which brings intracellular kinase domains into close proximity for *trans*-autophosphorylation to initiate downstream signaling cascades. According to the “rotation model”, ligand binding to the extracellular domain of the EGFR dimer induces rotation of the transmembrane domains parallel to the plane of the cell membrane, which leads to the reorientation of the intracellular kinase domain dimer from a symmetric inactive configuration to an asymmetric active form. This model is also able to explain how oncogenic mutations activate the receptor in the absence of the ligand, without assuming that the mutations induce receptor dimerization. In this review, we discuss the mechanisms underlying the ligand-induced activation of the preformed EGFR dimer, as well as how oncogenic mutations constitutively activate the receptor dimer, based on the rotation model. Excellent reviews have recently been published on the mechanism of activation of EGFR based on the ligand-induced dimerization model [25,26,27,28,29].

## 2. Mechanism for Activation of EGFR by Ligand Binding

### 2.1. EGFR Has a Dimeric Structure

The epidermal growth factor receptor was one of the first receptors for which ligand-induced dimerization was proposed as the molecular mechanism of transmembrane signaling [30,31]. In this “ligand-induced dimerization model,” EGFR is thought to exist in monomeric form at the cell surface prior to ligand binding. Ligand binding to its extracellular domain induces dimerization of the receptor monomer, as a result of which intracellular kinase domains become closer and *trans*-autophosphorylate one another in the dimeric state. This model is based on evidence that detergent-solubilized EGFR molecules are detected as chemically cross-linked dimers in the presence of a bound ligand, while in the absence of the ligand, a majority of the receptor is present in monomeric form. Similar results are also obtained by the chemical cross-linking of EGFR expressed in living cells [32]. Furthermore, a modified model has proposed that the receptor monomers are at equilibrium with receptor dimers [33,34]. A minor fraction of the receptor (<10% of total receptors) exists in dimeric form, in which its cytoplasmic kinase domains are compatible with *trans*-autophosphorylation (active dimer). Ligand binding to the extracellular domain shifts the equilibrium toward the formation of active dimers and induces kinase stimulation [33,34,35].

However, numerous studies have demonstrated that prior to ligand binding, EGFR exists in a dimeric, yet inactive, form at the cell surface [8,36]. By using different chemical cross-linkers from those previously employed, it is found that EGFR exists in dimeric form prior to ligand binding [37,38]. Hetero-Förster resonance energy transfer (FRET) [39,40,41], fluorescence correlation spectroscopy [39,40,41,42,43,44], and homo-FRET [45,46] analyses also demonstrate that EGFR exists in dimeric form at physiological expression levels. Single-molecule observation with total internal reflection fluorescence (TIRF) microscopy using oblique illumination also indicates the existence of dimers in living cells [47]. Fluorescent protein fragment complementation demonstrates that EGFR molecules at the cell surface are present in dimeric form, and that the addition of EGF to the cell culture does not increase the fluorescent intensity of the cells [48]. In this experiment, it is also shown that the homodimeric EGFR molecules are inactive, since EGF binding induces the phosphorylation and internalization of the receptor dimer. The results of reversible firefly luciferase enzyme fragment complementation [49,50,51] and homo-FRET [45] analyses demonstrate that EGFR exists in dimeric form at the cell surface, since no fluorescence intensity increase is observed upon the addition of EGF to the cell culture. Consistently, treatment of EGFR-expressing cells with inhibitors of protein tyrosine phosphatases induces EGFR phosphorylation at the same level as in cells stimulated with EGF [52]. All these results demonstrate that prior to ligand binding, 100% of the EGFR molecules are present in a dimeric form in living cells.

**Figure 2:**
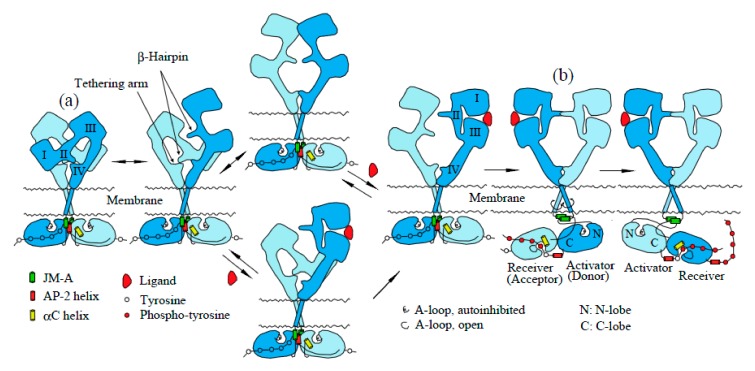
Model for the activation of EGFR by ligand binding. EGFR exists in dimeric form, stabilized through the interaction of the intracellular domains, the transmembrane domains, and the extracellular C-terminal regions of Subdomain IV. The intracelluar kinase domains with a symmetric configuration are further stabilized through interactions with the AP-2 helix. The extracellular domain of the receptor dimer adopts flexible configurations between “tethered” and “extended” forms through the interaction of Subdomains II and IV. The ligand has high affinity for the “extended” structure, and stabilizes the extracellular ligand binding domain for exposure of the “β-hairpin.” Interaction between the two “β-hairpins” in the dimer induces rotation of the transmembrane domains, which dissociates the intracellular symmetric inactive kinase dimer, resulting in an asymmetric active kinase dimer. In this asymmetric active configuration, the C-lobe of the “activator” kinase domain interacts with the N-lobe of the “receiver” kinase for the activation of the latter. Note that structures (**a**) and (**b**) indicate a tethered inactive dimer and an extended active dimer, respectively.

The existence of preformed EGFR dimers has recently been challenged. FRET analysis of EGFR fluorescently tagged at its N-terminus suggested that a majority of EGFR molecules were present in monomeric form [53]. As described below, the extracellular domain of EGFR adopts a tethered (closed) or extended (untethered or open) conformation in the absence of a bound ligand, while ligand-bound EGFR only has an extended structure (Figure 2). Therefore, FRET between fluorophores tagged at the N-terminus of EGFR does not correctly report whether EGFR is monomeric or dimeric, since FRET is dramatically changed before and after ligand binding. As described above, FRET between fluorophores tagged at the C-terminus of EGFR demonstrates the existence of preformed EGFR dimers in the absence of a bound ligand [39,40,46].

Using spectrally distinct quantum dots (Qdots) conjugated to either EGF or a monovalent Fab antibody, the transition between monomeric and dimeric EGFR was observed [54,55]. This transition is likely to occur between EGFR dimers and tetramers, since only one Fab molecule conjugated with a Qdot seems to bind EGFR dimers because of its large size. Two Fab molecules conjugated with a Qdot are unlikely to be able to bind an EGFR dimer. Furthermore, two EGF molecules conjugated with a Qdot cannot bind to an EGFR dimer because of negative cooperativity, as described below. Therefore, a Qdot-conjugated EGF or Fab is likely to report EGFR dimers as monomers. Upon activation, the EGFR dimers become tetramers and oligomers through the interaction between EGFR itself [56,57], as well as the interaction with its effectors such as AP-2 and Shc1. This complex seems to interact with actin filaments for clustering while it moves toward sites for clathrin-dependent and -independent endocytosis [10].

More recently, EGFR-eGFP expressed in *Xenopus* oocytes at low levels, ~1 to ~5 molecules per µm^2^, existed in monomeric form when analyzed by photobleaching [56]. This is inconsistent with previous results in which the EGFR expressed in CHO cells at low levels, ~3 molecules per µm^2^, was present in dimeric form [43]. In fact, the dimers are formed in the endoplasmic reticulum before reaching the cell surface [48], and the dimer formation does not depend on the expression levels of the receptor [39,43,45]. These results indicate that EGFR has a stable dimeric structure, which does not dissociate. To confirm the EGFR monomer, it is therefore necessary to observe whether two EGF-bound monomeric EGFR molecules merge to form dimers on the surface of *Xenopus* oocytes, using a TIRF microscope.

### 2.2. Structures of Inactive and Active EGFR Dimers

Crystal structures of the extracellular domain of unliganded and ligand-bound EGFR have revealed large conformational changes [14,15,58,59]. An intramolecular tether is observed in the extracellular domain of unliganded EGFR (Figure 2a). The “β-hairpin” of Subdomain II is buried and interacts with a “tethering arm” (also called “C1 modules” or “a pocket”) at the C-terminal end of Subdomain IV to form an “auto-inhibited” conformation [58,60,61]. In the ligand-bound form, Subdomains I and II rotate and move away as a unit from Subdomain IV so as to be stabilized in an extended conformation in which the “β-hairpin” of Subdomain II and the “tethering arm” of Subdomain IV are positioned to interact one another to form a back-to-back, dimeric complex (Figure 2b) [14,15]. Each ligand molecule is clamped between Subdomains I and III of the same EGFR protomer. The ligand-free “tethered” conformation and the ligand-bound “extended” conformation are mutually exclusive. Furthermore, based on both biochemical studies [62] and modeled structures [58], Subdomain IV is also thought to directly contact the dimer interface to form an inactive “tethered” dimer prior to ligand binding.

Structures of the EGFR kinase domain in a symmetric inactive dimer [63] and an asymmetric active configuration [64] provide insight into the conformational changes of the kinase dimer during activation. In the symmetric inactive dimer, helix αC of the kinase N-lobe rotates outward with respect to its conformation in the active state. The activation loop (A-loop) is tightly packed inside the active site in a way that blocks the binding of peptide substrates (Figure 2). Furthermore, the symmetric inactive kinase dimer is stabilized by the AP-2 helices, which interact with interfaces of two protomers of the dimer (Figure 2a). This helix is a recognition element in EGFR by the AP-2 clathrin adaptor protein [65]. An “electrostatic hook”, which consists of acidic side chains (D1003, E1004 and E1005; D979, E980, and E981 in mature EGFR) in the turn after the AP-2 helix, forms ion pairs with residues in the kinase domain (H773, H850, K852 and K846; H749, H826, K828, and K822 in mature EGFR) [63]. In the asymmetric active kinase domain dimer, the C-lobe of the “activator” (also called the “donor”) kinase contacts the N-lobe of the adjacent “receiver” (also called the “acceptor”) kinase, and promotes conformational changes that activate the “receiver” kinase (Figure 2b) [63,66]. Thus, ligand binding to the extracellular domain of the EGFR dimer is likely to dissociate the symmetric inactive kinase dimer, and to reorient it to the asymmetric active kinase dimer in which the “activator” kinase induces a conformational change of the adjacent “receiver” kinase. Upon activation, helix αC rotates toward the active site, inducing an open conformation of the A-loop that allows substrate binding [63,64,67]. In the asymmetric active dimer, the N-terminal part of the intracellular JM region (referred to as JM-A; Figure 1 and Figure 2) of the “receiver” and “activator” kinases is likely to interact so as to form an anti-parallel coiled coil structure (Figure 2b) [63]. The C-terminal part of the JM region (referred to as JM-B or JMAD) of the “receiver” kinase interacts with the C-lobe of the “activator” kinase domain to function as a latch for stabilizing the active configuration [63,66].

### 2.3. Negative Cooperativity in Ligand Binding to EGFR

Scatchard analysis of EGF binding to EGFR at the cell surface yields concave-up or curvilinear plots that indicate the presence of two classes of binding sites. It has long been thought that the two classes would be EGFR monomers and dimers with low and high affinity, respectively [68,69]. However, the heterogeneity in EGF-binding affinities has recently been shown to arise from negative cooperativity in the ligand-receptor interaction [70,71], which had been previously predicted [72]. Similarly, the analysis of insulin binding to the insulin receptor also reveals curvilinear (concave-up) Scatchard plots due to a negative cooperative interaction between insulin and its receptor, which has a covalently cross-linked dimeric structure with cysteine disulfide bridges [73]. Therefore, it is not necessary to assume the existence of a monomeric form of EGFR at the cell surface.

Crystal structures of the extracellular domain of *Drosophila* EGFR also suggest negative cooperativity in its ligand binding [74]. The negative cooperativity requires that the receptor dimer binds one ligand, and the binding of a second ligand must occur with a lower affinity than the first. Consistent with this, single ligand binding seems to be sufficient to activate the EGFR dimer [75]. There is evidence that the interaction between the “tethering arms” (Residues 585–609 of Subdomain IV; 561–585 in mature EGFR) in the extracellular domains of the EGFR dimer is essential for negative cooperativity in EGF binding [76]. Breaking the disulfide bond between C582 and C591 (C558 and C567 in mature EGFR) of the “tethering arm” of Subdomain IV through a double alanine replacement entirely abrogates the negative cooperativity. The deletion of a loop between C595 and C617 (C571 and C593 in mature EGFR) of the “tethering arm” also has the same effect [76]. These results demonstrate that the “tethering arm” plays a vital role in supporting negative cooperativity in ligand binding, and they also suggest that the “tethering arm” contributes to an inter-subunit interaction within the EGFR dimer prior to ligand binding.

The intracellular JM region is divided into two segments, JM-A and JM-B (Figure 1) [63,66]. NMR and mutational analyses of JM-A suggest that these segments form an anti-parallel helical dimer (Figure 2b) [63]. The deletion of JM-A results in the complete loss of negative cooperativity in ligand binding to EGFR [77]. In the anti-parallel helical dimer of JM-A segments in the receptor dimer, E687 and E690 (E663 and E666 in mature EGFR) are predicted to be involved in salt bridges that stabilize the dimeric structure. When these ionic interactions are removed by mutation, the negative cooperativity is abrogated [77]. This again suggests that the proposed anti-parallel helical dimer contributes to the negative cooperativity. It has long been recognized that the phosphorylation of EGFR T678 (T654 in mature EGFR) leads to a decreased affinity of EGF and the loss of EGF-stimulated receptor autophosphorylation [78,79,80,81,82]. Phorbol 12-myristate 13-acetate-induced phosphorylation of T678 by protein kinase C leads to the complete loss of cooperativity [77]. These results also suggest that the JM-A segment plays a role in negative cooperativity in ligand binding to the EGFR dimer. Considering all of the results described above, EGFR exists in dimeric form in living cells prior to ligand binding.

### 2.4. Mechanism of Activation of EGFR Dimers by Ligand Binding: The “Rotation Model”

How is the EGFR dimer activated by ligand binding? It is known that the EGFR dimer is formed through an interaction of the intracellular domain [38,48], the transmembrane domain [83,84], and the extracellular C-terminal region of Subdomain IV [58,62]. Indeed, the crystal structures of the intracellular domain show that the kinase domain dimer adopts a symmetric, back-to-back, inactive structure, stabilized by the AP-2 helices (Figure 2a) [63]. Ligand binding to the extracellular domain of the EGFR dimer induces ~140° rotation of the transmembrane domain, parallel to the plane of the cell membrane [37], as observed in many other cell-surface receptors [36,85,86]. This rotation of the transmembrane domain is supported by an NMR structural study showing that the transmembrane domain of EGFR has two surfaces through which the domains interact with each other [87]. The two surfaces are located at opposite sides of the α-helix, ~140° apart from each other about the long axis. Upon ligand binding to an untethered extracellular domain of the receptor dimer, the domains assume an extended conformation, stabilized through the interaction of two exposed “β-hairpins” (Figure 2b). This conformational change, from tethered to extended, is likely to induce a ~140° rotation of the transmembrane domains, which reorients the symmetric inactive kinase domain dimer to the asymmetric active configuration. In the asymmetric active kinase dimers, the “activator” kinase induces a conformational change of the adjacent “receiver” kinase for activation, as described above.

It has been proposed that dimer-to-tetramer transition is required for the full activation of the receptor, in which two dimers become juxtaposed in a side-by-side arrangement [40,88,89]. Ligand-induced EGFR tetramerization through the interaction of a region within Subdomain IV of the extracellular domain seems to be essential for full activation of the receptor [56,57]. In this model, the phosphorylation of tyrosine residues in the C-terminal tail, proximal to the kinase domain, is facilitated by a continuous array of “activator” and “receiver” kinase domains of the receptor tetramers and oligomers. For this oligomerization, it is known that the receptor’s kinase activity and phosphorylation of substrate tyrosine residues are required [45,48]. Therefore, ligand binding first activates the receptor dimer, and then the resulting phosphorylated dimers may assemble into oligomers for full activation. As discussed above, this transition seems to occur between EGFR dimers and tetramers [54,55].

As described above, EGFR has seven distinct ligands, which are capable of inducing different biological effects in the same cell [20]. For example, TGFα and AREG stimulate the proliferation of 32D cells more efficiently than EGF and HB-EGF, and these efficiency differences have been correlated with the differential phosphorylation of specific sites on the C-terminal tail of EGFR [90,91,92]. This is partly due to the different binding affinities of the distinct ligands for EGFR [93,94]. According to the proposed mechanism here, ligand binding to the extracellular domain of EGFR induces rotation of the transmembrane domain to dissociate and reorient the symmetric inactive kinase dimer to the asymmetric active dimer. How do the distinct ligands induce the differential phosphorylation of specific sites of EGFR? The differential phosphorylation may occur during tetramerization and oligomerization, the stability of which may be dependent on the affinity of bound ligands for EGFR. As described above, tetramerization and oligomerization are required for full activation of the receptor, in which two dimers become juxtaposed in a side-by-side arrangement [40,88,89]. Therefore, the stability of the tetramers and oligomers may affect the phosphorylation of specific sites of the receptor.

## 3. EGFR and Cancer

The epidermal growth factor receptor plays crucial roles in cellular processes such as cell proliferation and differentiation, and its aberrant activation is implicated in a variety of human cancers [5,95]. There are a number of mechanisms by which the tight regulation of EGFR signaling can be abrogated, including the enhanced production of ligands, overproduction of the EGFR protein, mutations leading to the constitutive activation of EGFR, deficiency of EGFR downregulation, and the activation of EGFR through cross-talk with heterologous cell-surface receptors [96]. In this review, we only focus on the overproduction of EGFR and mutations causing constitutive activation of EGFR. Constitutive activation of EGFR by the overexpression and oncogenic mutation of the receptor have traditionally been explained by dimerization of the receptor monomers. Here, we explain the mechanisms of the constitutive activation based on the “rotation model,” without assuming that the mutations induce receptor dimerization.

### 3.1. EGFR Overproduction

The aberrant activation of EGFR is often associated with the overexpression of the *EGFR* gene [97,98]. Enhanced EGFR levels have been correlated with poor prognosis in various cancers including head and neck, bladder, ovarian, cervical, and esophageal cancers [99]. Amplification of the *EGFR* gene is one way to overproduce EGFR, and has been observed in various cancers including breast carcinomas [100], non-small-cell lung cancer (NSCLC) [101], and glioblastoma multiforme (GBM) [102,103]. In the absence of gene amplification, EGFR can also be overproduced at the level of transcription. It has been shown that wild-type and mutant p53 proteins directly activate *EGFR* transcription by specific binding to its promoter [104,105]. A specific region of *EGFR* has an enhancer activity in breast cancer cell lines overproducing EGFR [106,107]. The number of polymorphic CA dinucleotide repeats in the intron 1 of *EGFR*, close to the enhancer region, seems to be involved in the regulation of *EGFR* transcription, since transcription declines with increasing numbers of CA repeats [108,109,110]. Furthermore, laryngeal papilloma cells, which are benign epithelial tumors caused by infection with the human papilloma virus, produce high levels of EGFR, presumably due to the enhanced recycling of the receptor back to the cell surface following EGF stimulation [111].

How does overexpression activate EGFR in the absence of a bound ligand? As described above, the inhibition of protein tyrosine phosphatases spontaneously activates EGFR in the absence of the ligand to the maximum level induced by EGF binding [52]. This indicates that EGFR autophosphorylates at a basal level, presumably due to the incomplete stability of the symmetric inactive kinase dimer, and the resulting phosphotyrosine residues are dephosphorylated by the phosphatases. Therefore, phosphorylation levels of EGFR are determined by a balance between EGFR and phosphatase activities. When EGFR is overexpressed, the phosphorylation levels of EGFR may exceed the capacity of the phosphatases, resulting in the enhanced activation of EGFR in the absence of the ligand.

### 3.2. Mutations that Constitutively Activate EGFR

Mutations in the *EGFR* gene that constitutively activate the receptor frequently occur in human cancers. Here, we review such mutations classified by their location in the extracellular domain, the intracellular JM region, the intracellular kinase domain, and the intracellular C-terminal tail of EGFR, and we discuss how these mutations constitutively activate the receptor. In this review, we only discuss mutations that have been found in cancer patients and that constitutively activate the receptor.

#### 3.2.1. Mutations in the Extracellular Domain

Mutations in the extracellular domain of EGFR are frequently observed in GBM, in which the amplification of the *EGFR* gene is particularly prominent [112,113]. This gene amplification often accompanies deletions or insertions. The EGFR type I mutant, EGFRvI, lacks most of the extracellular domain, and is tumorigenic (Figure 1) [112]. EGFRvII has an in-frame deletion of exons 14 and 15, which encode amino acid residues 545–627 (521–603 in mature EGFR) of Subdomain IV [114]. The constitutive phosphorylation of EGFRvII at a level similar to that of wild-type EGFR stimulated with EGF was observed in serum starved cells, demonstrating ligand-independent activation [115]. The region deleted in EGFRvII encodes the “tethering arm,” which interacts with the “β-hairpin” of Subdomain II to form the inactive “tethered” conformation of the extracellular domain. Therefore, the EGFRvII deletion forces the mutant receptor to become the active extended form. EGFRvIII (also known as de2–7 EGFR or D2–7EGFR) has an in-frame deletion of exons 2–7, which encode amino acid resides 30–297 (6–273 in mature EGFR) encompassing Subdomain I and two-thirds of Subdomain II [112]. Despite the lack of ligand binding, EGFRvIII is constitutively phosphorylated, and confers high tumorigenic potential [116]. Unlike ligand-activated wild-type EGFR, however, EGFRvIII also activates the c-Jun N-terminal kinase through PI3K, but cannot activate the signal transducers and activators of transcription 1 (STAT1) and STAT3 [98,117,118]. EGFRvIII fails to be ubiquitinated, thus prolonging oncogenic signaling [119].

How are these mutants with deletions in the EGFR extracellular domain constitutively phosphorylated? As described above, the pre-formed dimeric structure of EGFR is facilitated through interactions between the cytoplasmic domains, the transmembrane domains, and the extracellular JM regions. Therefore, EGFR mutants with deleted extracellular ligand-binding domains are able to adopt a dimeric form, and are likely to *trans*-autophosphorylate. This suggests that the extracellular domains of the EGFR dimer impose a negative constraint on receptor activation that is relieved by EGF binding. In the absence of the N-terminal extracellular regions, therefore, the cytoplasmic domain dimer of the deletion mutants may be unstable, and the kinase domain dimer tends to adopt an asymmetric active configuration. However, this active configuration does not seem to be the same configuration as that of ligand-activated wild-type EGFR. Only limited numbers of substrate tyrosine residues seem to be phosphorylated [120]. This may cause abnormal downstream signaling and the lack of ubiquitination. The extracellular deletions may also induce the rotation of the transmembrane domains, which then rearranges the kinase domain dimer for partial activation. Indeed, EGFRvIII spontaneously forms cysteine disulfide-bridged dimers that are phosphorylated in the absence of the ligand [121]. The cysteine disulfide bridging of the EGFRvIII receptor in the dimeric form may induce the rotation of its transmembrane domains, resulting in the reorientation of the intracellular kinase domains from a symmetric inactive form to an asymmetric active configuration. However, the mechanism of activation of EGFRvIII must await determination of its structure.

Missense mutations (R108K, T263P, A289V and G598V; R84K, T239P, A265V, and G574V in mature EGFR) in the extracellular domain of EGFR were also found in GBM and glioblastoma cell lines, which conferred anchorage-independent growth and tumorigenicity upon NIH 3T3 cells [113,121,122,123]. These mutant EGFR proteins are phosphorylated at basal levels, and are still responsive to the ligand. R108K and A289V occur at the interface between Subdomains I and II, and T263P occurs in Subdomain II just before the “β-hairpin” that contacts Subdomain IV in the inactive “tethered” structure (Figure 2a). These mutations may cause conformational changes of the extracellular domain of EGFR, and may release the tether between Subdomains II and IV. The released “β-hairpin” of Subdomain II may interact eith each other to induce the ligand-independent basal phosphorylation of the EGFR missense mutants (Figure 2b). The detailed mechanism of activation of the mutants must await determination of their structures.

Sequencing of EGFR in a large cohort of GBM patients has identified over 30 different missense mutations within the extracellular domain, including C620Y, C620W, C624F, C628Y, and C636Y (C596Y, C596W, C600F, C604Y, and C612Y in mature EGFR). All of these cysteines are present in Subdomain IV [113,124,125,126]. These Subdomain IV mutations leave an unpaired cysteine residue available for the formation of an intermolecular disulfide bond. Unlike EGFRvIII, which spontaneously formed a disulfide-bridge between protomers, these mutants form interprotomer cysteine disulfide bridges upon ligand stimulation [121]. Three of the four mutants (C620Y, C624F, and C628Y) exhibit elevated levels of basal-tyrosine phosphorylation, with C620Y having the highest level. In particular, cell lines expressing the C620Y or C624F mutant grew better in soft agar than the control cells expressing wild-type EGFR, suggesting that these mutations are oncogenic [121]. An EGFR-ligand autocrine loop may initiate the activation of the mutant receptors, and may then induce disulfide bridge formation that in turn rotates the transmembrane domains to constitutively activate the mutant receptors. This proposed mechanism of activation of the mutants must await determination of their structures before and after ligand binding.

#### 3.2.2. Mutations in the Intracellular JM Region

V689M and L703F (V665M and L679F in mature EGFR), found in NSCLC patients, constitutively activate the mutant EGFR in the absence of a bound ligand to the same level as the wild type stimulated with a ligand [66]. A possible structural basis for the EGFR activation by the V665M or L679F mutation is that the side chain of a methionine substituted V689 projects into a cavity on the surface of the C-lobe, while the V689 side chain does not fill this cavity [66]. This interaction between the side chain of the methionine and the cavity of the C-lobe may stabilize an asymmetric active structure of the kinase domain dimer. The mutation of L703 to phenylalanine could improve the packing of the JM-B of the “receiver” kinase with the C-lobe of the “activator” kinase for stabilization of the active configuration [66]. The mutations may also stabilize the asymmetric active dimeric form when spontaneous transition from a symmetric inactive dimer to an asymmetric active form occurs, because the symmetric inactive structure does not seem be completely stable. Again, the detailed mechanism of the activation of the mutants must await mutagenic analysis of the mutants and ultimately the determination of their structures.

#### 3.2.3. Mutations in the Kinase Domain

The G719S mutation (G695S in mature EGFR) also activates the kinase, presumably by the destabilization of the symmetric inactive configuration of the dimer [127,128,129]. G719 contributes to interactions that hold helix αC in the inactive conformation [130]. The G719S mutation may not be able to do this, and may allow the helix to adopt its active conformation in the asymmetric dimeric configuration. Substitutions of G719 to alanine or cysteine also occur in NSCLC [131,132]. Structural studies of the mutants are required to test this mechanism of activation.

Deletion mutants in the intracellular domain have also been found in NSCLC, and various deletions within the range encompassing E746 to I759 (E722 to I735 in mature EGFR) have been identified [133]. A deletion mutant, ∆E746–A750 (∆E722–A726 in mature EGFR), is constitutively phosphorylated in the absence of the ligand [134]. Two other mutations are a deletion in which residues from L747 to P753 (L723–P729 in mature EGFR) are replaced with a single serine to give the ∆L747–P753insS mutant [133,135,136], and the mutant ∆S752–I759 (∆S728–I735 in mature EGFR) in which residues 752–759 are deleted [133]. When these mutant *EGFR* genes were expressed in the murine hematopoietic Ba/F3 cell line, they all showed significantly higher basal (ligand-independent) tyrosine phosphorylation levels than wild-type receptors [128]. The regions deleted from the N-terminus of helix αC in the mutants interact with the A-loop in inactive EGFR [64]. This suggests that the removal of the region by deletion may activate EGFR by disrupting the autoinhibited kinase domain conformation through the release of the A-loop from the active site for substrate binding [128]. This conformational change of the active site of EGFR may cause the transition from a symmetric inactive configuration to an asymmetric active form in the dimer. The detailed mechanism of activation of the mutants must await determination of their structures.

The R776H mutation (R752H in mature EGFR) is associated with lung cancer where there is no smoking history, and is found both in normal and tumor tissues [137]. This mutant receptor is constitutively autophosphorylated in the absence of the ligand [137,138]. The constitutive activation of the receptor relies on the intact interface in its asymmetric dimeric kinase configuration. The R776H mutant receptor preferentially adopts the “receiver” position when paired with wild-type EGFR [138], providing support for the “superacceptor” hypothesis [139,140]. This is similar to the L858R mutation described below.

G735S, G796S, and E804G mutations (G711S, G772S, and E780G in mature EGFR) found in prostate cancer are oncogenic, causing increased cell growth, transformation, and invasion [141]. All of these mutant receptors demonstrate the increased phosphorylation of four tyrosine residues (Y869, Y1016, Y1092, and Y1197; Y845, Y992, Y1068, and Y1173 in mature EGFR) compared to wild-type EGFR in the absence of the ligand, indicating that the mutants are constitutively active. The G735S and G796S mutations were also found in thyroid cancer and squamous cell carcinoma of the head and neck, respectively [142,143]. The G735 residue is located in the N-lobe, and this mutation is likely to cause a conformational change of the kinase domain for activation [142]. G796 and E804 are located at the interface between two protomers of a symmetric inactive dimer, and seem to be involved in inactive dimer formation. The G796S and E804G mutations may destabilize the inactive dimer to form an asymmetric active configuration. It is necessary to test this proposed mechanism by observing the effects of mutagenesis of the residues and interacting residues on the autophosphorylation of the mutant receptors.

The kinase domain mutation L858R (L834R in mature EGFR) has been observed in 40–45% of mutations in NSCLC [144]. An asymmetric configuration of the kinase domain dimer is required for the ligand-independent activity of NSCLC-associated EGFR kinase mutants [140,145]. Crystal structural analysis indicates that the L858R mutation is likely to shift the equilibrium of the kinase domain dimer toward an active configuration by preventing key hydrophobic interactions between L858 and other residues in the N-lobe that lock the regulatory helix αC in the inactive position [128,130]. This occurs by suppressing intrinsic disorder within the kinase domain N-lobe to allow the mutant kinase domain to act as a “superacceptor” within asymmetric kinase dimers [139,140]. In this ligand-independent activation of EGFR due to L858R, “β-hairpin” interaction of the extracellular Subdomain II plays a critical role [54]. Interestingly, live-cell FRET measurements reveal that the L858R kinase mutation alters the extracellular domain structure such that unliganded mutant EGFR adopts an extended (untethered) configuration. Therefore, the transition from a symmetric inactive configuration of the kinase dimer to an asymmetric active form affects configurations of the extracellular domains, causing the “β-hairpins” to interact. This inside-out transmembrane signaling may occur through a rotation of the transmembrane domains. To test this possibility, a structural study of the mutant is required.

The L861Q mutation (L837Q in mature EGFR) found in NSCLC is also in the A-loop of the kinase domain [122]. A short α-helix in the A-loop that includes residues L858 and L861 interacts with helix αC in the inactive form of EGFR. Side chains of both L858 and L861 contribute to a hydrophobic core formed by residues from the helix αC, the A-loop, and elsewhere [64,146]. The L861Q mutation could also disrupt the autoinhibitory interaction between the A-loop and helix αC of EGFR by destabilizing the set of hydrophobic interactions [128]. Therefore, L861Q is likely to shift the equilibrium of the kinase domain toward an active conformation, which acts as a “superacceptor” within asymmetric kinase dimers.

An in-frame, tandem duplication of EGFR residues 688–1054 (664–1030 in mature EGFR) has been detected in GBM [113,147]. This duplication region comprising exons 18–26 encodes the entire kinase domain, and the mutant designated “TKD-EGFR” displays chronically elevated basal autophosphorylation at five known phosphorylation sites [148]. An EGFR kinase domain duplication has also been found in lung, brain, and other cancers [149]. This mutant, EGFR-KDD, has a tandem, in-frame duplication of exons 18–25, which encode the entire EGFR kinase domain, and is constitutively active. Computational modeling suggests that EGFR-KDD can be activated by virtue of an asymmetric intramolecular dimeric structure, unlike the typical asymmetric, intermolecular dimeric structure between adjacent protomers [149].

#### 3.2.4. Mutations in the C-Terminal Tail Region

As described above, a large fraction of GBM cells display *EGFR* gene amplification that correlates with receptor overexpression [102], and also exhibit an EGFRvIII deletion mutation in the extracellular domain of the receptor [150]. EGFR molecules with C-terminal deletions have also been found in GBM, and are collectively termed EGFRvIV. EGFRvIVa lacks exons 25–27 (from G983 to P1090; from G959 to P1066 in mature EGFR), and EGFRvIVb lacks exons 25 and 26 (from G983 to G1054; from G959 to G1030 in mature EGFR) [113,151,152]. These two mutants have transforming and tumorigenic properties in the absence of the ligand, and show ligand-independent constitutive activation. The oncogenic function of these mutants depends upon the intrinsic kinase activity [153]. Recent genomic studies in GBM and NSCLC patients have identified an additional class of oncogenic mutations caused by the deletion of C-terminal coding regions [103,154,155,156]. Among them, mutants with a deletion of exon 25 (from G983 to L1038; from G959 to L1014 in mature EGFR) or both exons 25 and 26 (from G983 to G1054; from G959 to G1030 in mature EGFR, which is equivalent to EGFRvIVb above) are constitutively phosphorylated, and cause phosphorylation of downstream STAT3 when expressed in NIH 3T3 cells in the absence of the ligand [156,157]. Exon 25 encodes 56 amino-acid residues from G983 to L1038 (from G959 to L1014 in mature EGFR), and encompasses the AP-2 helix and the electrostatic hook, which are involved in the stabilization of the symmetric inactive configuration of the kinase dimer [63]. Therefore, it is likely that the deletion of exon 25 destabilizes the inactive kinase dimer, and shifts the equilibrium of the kinase domain toward an asymmetric active configuration in the dimeric structure. The detailed mechanism of activation of the mutants must await determination of their structures.

## 4. Conclusions

It is now clear that EGFR is present in an inactive dimeric form at the cell surface in the absence of the ligand. Binding of the first ligand to one of the subunits lowers the affinity of the second to the other subunit in its dimeric structure (negative cooperativity). Ligand binding to the extracellular domain of the receptor dimer stabilizes the extended conformation of the domains, which interact through Subdomain II. This interaction seems to induce the rotation or twist of the transmembrane domains of the receptor dimer, which reorients the intracellular kinase domain dimer from a symmetric inactive configuration to an asymmetric active dimer (the “rotation model”). EGFR is also an allosteric enzyme, and one of the kinase domain dimers activates the other in the asymmetric configuration.

This molecular mechanism for the activation of EGFR explains how oncogenic mutations spontaneously activate the receptor in the absence of a bound ligand, without assuming that such mutations induce the dimerization of receptor monomers. Many of the mutations are likely to destabilize the symmetric inactive dimeric structure of the intracellular domains and to rearrange the domains into the asymmetric active dimeric configuration. The rotation of the transmembrane domains for the regulation of EGFR activity is also able to explain the inside-out transmembrane signaling, and presents new opportunities for the design of anti-cancer drugs.
